# RecA-SSB Interaction Modulates RecA Nucleoprotein Filament Formation on SSB-Wrapped DNA

**DOI:** 10.1038/s41598-017-12213-w

**Published:** 2017-09-19

**Authors:** Hung-Yi Wu, Chih-Hao Lu, Hung-Wen Li

**Affiliations:** Department of Chemistry, National Taiwan University, Taipei, Taiwan

## Abstract

*E*. *coli* RecA recombinase catalyzes the homology pairing and strand exchange reactions in homologous recombinational repair. RecA must compete with single-stranded DNA binding proteins (SSB) for single-stranded DNA (ssDNA) substrates to form RecA nucleoprotein filaments, as the first step of this repair process. It has been suggested that RecA filaments assemble mainly by binding and extending onto the free ssDNA region not covered by SSB, or are assisted by mediators. Using the tethered particle motion (TPM) technique, we monitored individual RecA filament assembly on SSB-wrapped ssDNA in real-time. Nucleation times of the RecA E38K nucleoprotein filament assembly showed no apparent dependence among DNA substrates with various ssDNA gap lengths (from 60 to 100 nucleotides) wrapped by one SSB in the (SSB)_65_ binding mode. Our data have shown an unexpected RecA filament assembly mechanism in which a RecA-SSB-ssDNA interaction exists. Four additional pieces of evidence support our claim: the nucleation times of the RecA assembly varied (1) when DNA substrates contained different numbers of bound SSB tetramers; (2) when the SSB wrapping mode conversion is induced; (3) when SSB C-terminus truncation mutants are used; and (4) when an excess of C-terminal peptide of SSB is present. Thus, a RecA-SSB interaction should be included in discussing RecA regulatory mechanism.

## Introduction

RecA nucleoprotein filaments are the repair-active species in the highly conserved homologous recombination (HR) DNA repair pathway in *E*. *coli* ^1^. In this pathway, double-stranded DNA breaks are first processed by the RecBCD helicase/nuclease into a partially single-stranded fragment. RecA then binds onto the single-stranded DNA (ssDNA) in the presence of ATP to form a helical nucleoprotein filament, with a net 5′-to-3′ assembly polarity^2^. This RecA nucleoprotein filament is the essential component to execute the different steps in HR repair process: searching homologous DNA, pairing homology DNA and catalyzing DNA strand exchange^1^. Thus, the assembly of a RecA nucleoprotein filament is subjected to tight regulation^3^. There exists an analogous pathway in eukaryotes and humans, and the formation of a recombinase nucleoprotein filament is the key step subjected to complex regulation^4–8^.

Dynamics of RecA filament assembly in the absence of other DNA binding proteins has been investigated by both conventional, ensemble-based biochemical experiments as well as various single-molecule ones^1,9–15^. These studies collectively define the kinetics, the rate-limiting step, the nucleation unit, assembly and disassembly polarity, as well as the rates of nucleation and extension steps. *In vivo*, ssDNA molecules are prone to nuclease degradation and environmental insults and are mostly protected and stabilized by binding to single-stranded DNA binding proteins (SSB) in *E*. *coli* ^16^. These single-stranded DNA binding proteins play central roles in DNA replication, recombination, and repair^16^. *E*. *coli* SSB binds onto ssDNA by wrapping ssDNA around the SSB homotetramers^17^. SSB exists with multiple DNA binding modes, the most prominent of which encompass either 35 nt or 65 nt^16–19^. Conversion among these different SSB binding modes depends on salts, tension on ssDNA substrates and free SSB concentrations^17–19^. For example, the (SSB)_65_ binding mode binds to ssDNA with very high binding affinity and moderate cooperativity^20^, but remains extremely mobile and diffuses along ssDNA^21–24^. The crystal structure shows that ssDNA wrapping on the whole protein resembles a “baseball seam” topology in the (SSB)_65_ binding mode^17^. In the (SSB)_35_ binding mode, SSB binds with high cooperativity onto ssDNA^17,25^. SSB also serves as an interaction platform for other proteins to process ssDNA^16,23^. For example, the *E*. *coli* PriA protein interacts with the SSB C-terminus to initiate replication restart^26^. RecO, an accessory protein in the HR process, also binds to the C-terminus of SSB to promote RecA loading onto ssDNA^23^. SSB facilitates unzipping of secondary structures such as hairpins during diffusion along ssDNA, thus allows loading of other ssDNA processing enzymes. Fluorescence imaging of dye-labeled individual SSB molecules also demonstrates the inter-segment transfer among different SSB binding modes^21^. The motion along ssDNA, the versatile dynamics of binding mode conversion and a wide range of protein-protein interactions make SSB a hub of ssDNA maintenance.

*E*. *coli* SSB and RecA, both as ssDNA binding proteins, compete for ssDNA substrates *in vivo*. In the presence of SSB, the DNA-dependent ATP hydrolysis rate of RecA declines, reflecting inhibition of RecA-ssDNA assembly^27,28^. Previous studies have suggested a passive nucleation model during RecA filament formation on SSB-wrapped ssDNA^2^, where RecA nucleation takes place in the presence of a transiently exposed, free ssDNA segment not covered by SSB. This model is supported by several observations: (i) RecA and SSB are both ssDNA binding proteins and their binding to ssDNA are mutually exclusive^2^, (ii) RecA nucleation onto SSB-wrapped ssDNA is significantly slower than onto the ssDNA without SSB^12^, and (iii) the RecFOR machinery substantially accelerates RecA assembly onto SSB-wrapped ssDNA^2^. Nevertheless, could a transient RecA-SSB interaction play a role in RecA assembly on the SSB-wrapped ssDNA? Notably, such an interaction between either HsRad51 or HsDmc1 has been demonstrated by co-IP^29^.

No apparent RecA-SSB interaction was identified by EM or pull-down assays^16,30^. Some RecA mutants bypass SSB inhibition, making them good choices for searching for a transient RecA-SSB interaction. For example, the RecA E38K mutation or a C-terminus deletion on EcRecA have been shown to bind to SSB-coated ssDNA much more rapidly than the wild type protein^27,31,32^. Interestingly, a combination of E38K and ΔC17 mutations showed further enhancement, suggesting the similarity and independence of these two mutations^27,32^.

Several fluorescence-based single-molecule studies attempted to address how RecA displaces SSB to form nucleoprotein filaments^2,12,18,24^. It takes RecA tens of minutes to displace SSB-wrapped ssDNA^2^, but the typical lifetime of single molecule fluorescence is limited to 3 minutes. Experimental modifications using either a pre-assembled RecA-ATPγS-dsDNA nucleus to significantly enhance the assembly process^12,24^ or taking only snapshots of dye-labeled RecA on SSB-bound kilo-nucleotide ssDNA^2^ were used. However, these approaches compromised either temporal (pre-assembled RecA-dsDNA filament) or spatial information (diffraction-limited spot of RecA nucleation cluster landing onto a ssDNA without defined numbers of bound SSB molecules) of how RecA initiates filament formation on SSB-wrapped ssDNA. A single-molecule tool with a time window of tens of minutes in real-time and a precise control of bound SSB numbers are required.

In this study, we monitored individual RecA filament formation by a single-molecule tethered particle motion (TPM) technique and by using DNA substrates with specified numbers of bound SSB. A RecA mutant (RecA E38K) displaying accelerated binding to SSB-coated ssDNA relative to wild-type RecA (wtRecA) is used to accelerate data acquisition^31^. Through the long-time acquisition, well-defined numbers of bound SSB and fast-reacting RecA mutant, it is possible to directly examine RecA filament assembly on SSB-ssDNA at the single-molecule level in real-time. Our work indicates the existence of a RecA filament assembly mechanism involving RecA-SSB interaction.

## Results

### SSB strongly inhibits RecA E38K filament assembly

In order to study the mechanism of how RecA protein assembles into nucleoprotein filaments, we modified the previously developed tethered particle motion (TPM) experiments^33,34^ by using DNA substrates containing a ssDNA gap region without secondary structure. For data acquisition purpose, we took advantage of a fast nucleating RecA mutant, RecA E38K. Previous studies suggest that it takes ~25 min for wtRecA to displace ~30% of the SSB from ssDNA, while RecA E38K can displace ~100% of the SSB in ~4 min at 42 °C^31^. More rapid binding of RecA E38K is also seen in our TPM experiments, carried out at 23 °C. Formation of wtRecA filaments on (dT)_90_ + SSB in 20 min at room temperature was essentially undetectable, while ~22% of the DNA substrates exhibited filament formation when RecA E38K was used (Figure S1G–I). In the absence of SSB, wtRecA assembled into filaments on about 4.5% of the DNA substrates within 2.5 minutes of observation, while 35% of the DNA tethers formed filaments with the E38K mutants (Figure S1A–F). We then directly assessed the assembly kinetics of wtRecA and RecA E38K using (dT)_90_ substrates in the absence of SSB. RecA E38K indeed displayed faster nucleation and extension time than wtRecA (Figure S1D,E). Nevertheless, the analogous BM increments observed showed that both E38K and wtRecA filaments are similar (Figure S1F).

**Figure 1:**
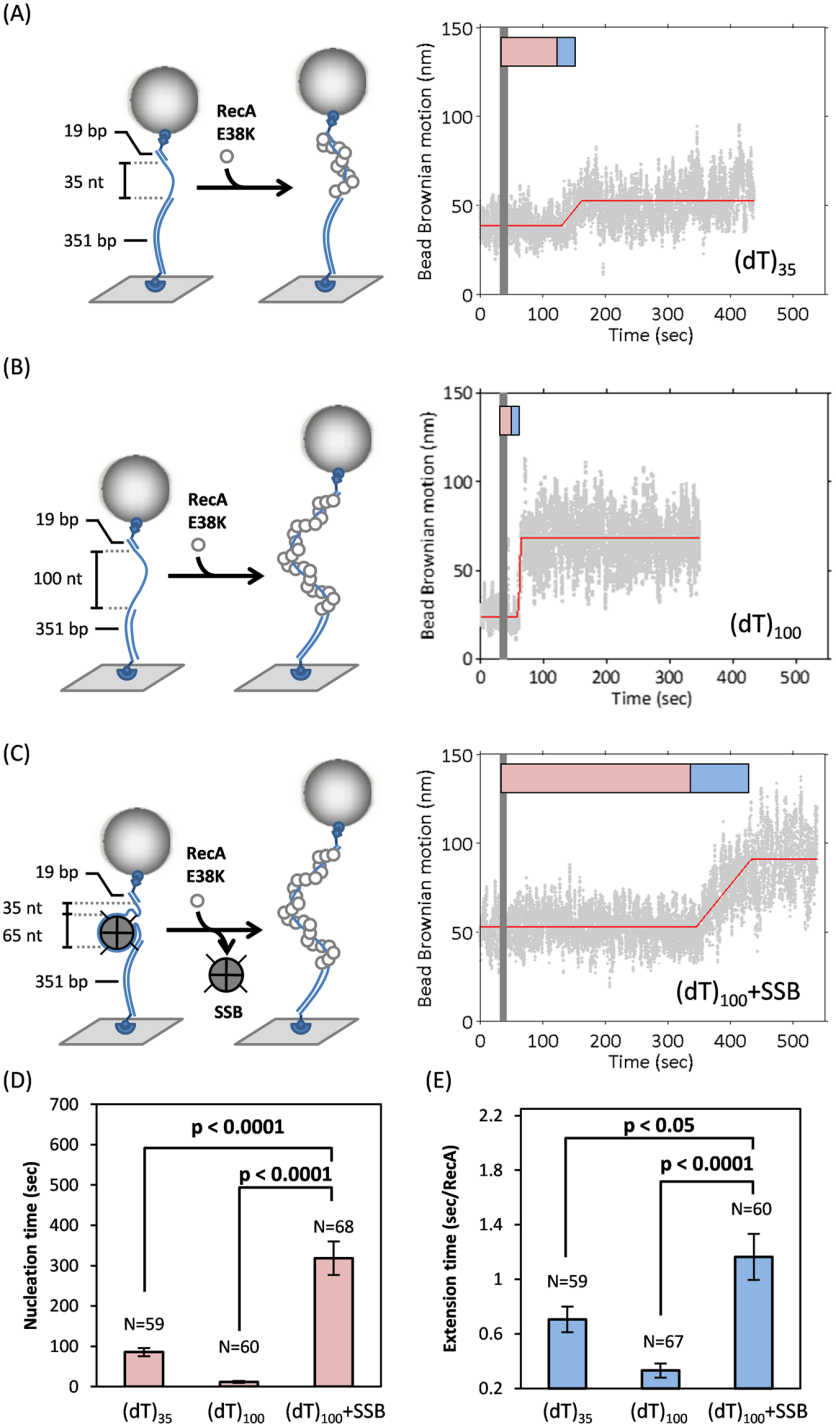
SSB inhibits RecA nucleoprotein filament assembly. (**A**–**C**) Schematic illustration of the experimental setup and representative bead BM time-courses of RecA E38K assembly on various DNA substrates: (**A**) (dT)_35_; (**B**) (dT)_100_ and (**C**) (dT)_100_ wrapped with a SSB tetramer. The reaction condition prepared the (SSB)_65_ binding mode as previously documented. RecA filament assembly leads to the apparent bead BM increase. (**D**) Mean nucleation time (second) and (**E**) mean extension time (second/RecA) of RecA E38K assembling on these DNA substrates. Error bar is one standard error of the mean.

**Figure 2:**
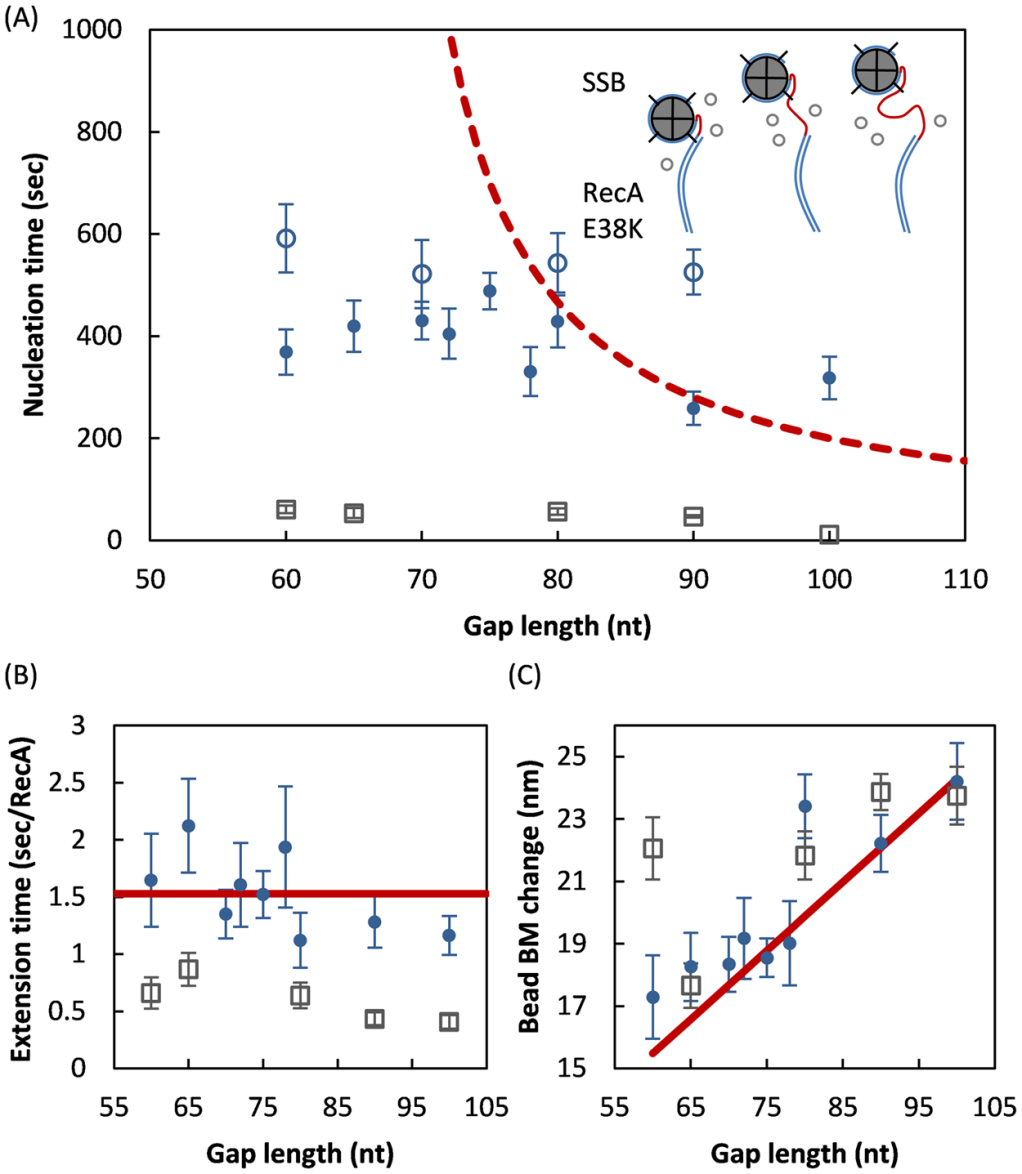
No apparent ssDNA length dependence of RecA E38K filament assembly kinetics on single SSB-wrapped ssDNA. (**A**) Mean nucleation time of RecA E38K on SSB-wrapped ssDNA-gap substrates ((dT)_n_, n = 60–100) shows no apparent difference on ssDNA lengths (filled blue circles). RecA E38K nucleation times on SSB-free substrates are significantly shorter (<100 seconds, gray open squares). The red dashed line is the prediction based on a pure passive nucleation mechanism with *τ* ^−1^ = *k*(*L* − 65) + *C*, where *τ*, *k*, *L*, 65 and *C* are mean nucleation time, microscopic rate constant, ssDNA gap length and number of nucleotides occupied by SSB in (SSB)_65_ binding mode, constant, respectively. Reactions were done in the presence of 2 μM E38K RecA. Using lower concentrations of E38K RecA (0.18 μM, blue open circles) leads to longer nucleation time, but also shows no ssDNA-length-dependence. (**B**) RecA mean extension time (sec/RecA) did not show variation with ssDNA length. In the presence of SSB, the extension time is slower. (**C**) Total bead BM change upon RecA filament assembly followed the expected trend. Lines are drawn for guidance purpose. Error bar is one standard error of mean.

To confirm that the presence of SSB strongly impairs RecA filament assembly, we prepared two ssDNA-gapped DNA substrates, containing either 35 or 100 nt poly(dT) (Fig. 1A,B). The double-stranded DNA handle in the ssDNA-gapped substrate is used to prevent the RecA-surface interaction in TPM experiments. Under the reaction condition used (pH = 7.5), RecA does not initiate the assembly process on the dsDNA region during the experimental time scale (~20 minutes, Figure S2). The (dT)_100_-containing DNA substrates were anchored in the reaction chamber, and then pre-incubated with excess SSB tetramers. The salt condition (10 mM Mg^2+^) used favors the (SSB)_65_ binding mode as previously suggested^12^. The (dT)_100_ substrate is stably bound by one SSB tetramer within the experiment time, considering the high binding affinity of SSB-ssDNA observed previously^23^. We then removed excess free SSB by buffer wash and introduced RecA E38K. Single-molecule TPM experiments monitor the RecA nucleoprotein filament assembly in real-time by measuring the bead Brownian motion (BM). Since RecA assembling on DNA leads to the stretching of DNA as well as a stiffness change, the bead BM increase directly monitors the nucleoprotein filament assembly. This single-molecule assay thus allows us to define the kinetic parameters during the assembly process, including nucleation time, the extension time and the final filament length, as previously shown^9,34^. We defined the dwell time of tethers between RecA introduction and significant bead BM increase (characterized by red blocks in Fig. 1A–C) as nucleation time, which represents the time required for RecA to form a stable nucleus on ssDNA. In this stage, bead BM did not change significantly even though there are several binding and dissociation events of few RecA monomers during the nucleation process. After stable nucleus formation, RecA rapidly polymerizes on ssDNA to form long, stable nucleoprotein filaments and results in significant bead BM increase (characterized by blue blocks in Fig. 1A–C). Earlier SSB work suggests that *E*. *coli* SSB in (SSB)_65_ binding mode stays bound onto ssDNA, but diffuses along ssDNA with a 1-D diffusion coefficient of 270 (nucleotides)^2^/sec(24). Therefore, the SSB on (dT)_100_ is likely to stay bound, but diffusive, as demonstrated in previous single-molecule work^21–24^. Because there is no free SSB in solution, the SSB binding mode is not changed^17^. Only up to 65 nt ssDNA should be covered by SSB in this (dT)_100_ substrate at all time, so the (dT)_100_ + SSB complex likely presents ~35 nt of free ssDNA at any given time. The other reaction with the (dT)_35_ substrate contains no SSB. We observed significant kinetic differences for the (dT)_35_ and (dT)_100_ + SSB substrates (Fig. 1A and C): delayed nucleation and slower extension for the (dT)_100_ + SSB substrates. Specifically, the mean nucleation times for RecA E38K binding to (dT)_35_ or (dT)_100_ + SSB are 85.6 ± 10.4 sec and 318.3 ± 41.6 sec, respectively. Nucleation times of RecA on SSB-free ssDNA substrates can be acquired by perfectly fitting the nucleation time histogram to a single-exponential decay^9,12,34^. In the case of SSB-wrapped ssDNA substrates, RecA nucleation times are not satisfactorily fitted to single-exponentials, likely reflecting multiple kinetic steps involved in the RecA-SSB-ssDNA interaction. Therefore, arithmetic mean of nucleation time is used for discussion, even though these values are likely underestimated. Based on the “passive-nucleation-only” model, RecA nucleation will occur only when a segment of ssDNA is transiently free from the SSB coverage, and is available for RecA to nucleate. In our TPM experiment, the (dT)_35_ and (dT)_100_ + SSB substrates should both contain ~35 nucleotides free ssDNA at any particular moment, yet the apparent nucleation times were significantly different. Considering the 35 nt ssDNA is longer than the required length implicated for the required nucleation unit of 2–6 RecA monomers^2,12^, the different nucleation times observed suggest a distinct RecA filament assembly mechanism in the presence of SSB (Fig. 1D). The mean extension times, expressed by the time required for adding one RecA (sec/RecA), are 0.71 ± 0.09 sec/RecA and 1.16 ± 0.17 sec/RecA for the (dT)_35_ and (dT)_100_ + SSB substrates, respectively. The slower mean extension time for (dT)_100_ + SSB suggests that during the filament extension process RecA encounters somewhat different molecular interactions in these two DNA substrates. Considering that the (dT)_100_ substrate is bound by SSB, this ~1.6-fold difference in extension time implies that SSB remains bound during RecA extension (Fig. 1E). We also performed RecA E38K assembly on SSB-free (dT)_100_ substrate as a control (Fig. 1B,D,E). This led to a much faster nucleation time (11.8 ± 2.27 sec) and extension time (0.41 ± 0.06 sec/RecA) compared to the (dT)_100_ + SSB reaction. These data support the idea that SSB strongly impedes RecA E38K filament assembly and indicates that SSB affects both the nucleation and extension steps. It’s possible that SSB could dissociate from ssDNA substrates during the RecA assembly reaction. However, we are certain that the (dT)_100_ substrate was bound by SSB (nucleation time of 318.3 ± 41.6 sec), since nucleation times of (dT)_60_-(dT)_100_ substrates were all less than 100 sec in the absence of SSB (Fig. 2A).

### Variation of ssDNA length does not change RecA E38K nucleation time

Since RecA E38K does not assemble on dsDNA under our experimental time scale and reaction conditions (Figure S2), RecA filament formation on a SSB-wrapped ssDNA has to occur either through binding directly to free ssDNA, or through interacting with the SSB-ssDNA complex (Fig. 1C). The observed nucleation time difference between SSB-free ssDNA and SSB-wrapped ssDNA (Fig. 1D) led us to speculate the relative contribution of these two competing steps. We prepared 10 gapped DNA substrates of different ssDNA lengths ((dT)_n_, n = 60–100) under experimental conditions in which a single SSB tetramer wraps some of the ssDNA in the (SSB)_65_ binding mode to study the effects of different amounts of unbound ssDNA on the RecA assembly process. These gapped DNA substrates are expected to expose excess ssDNA in a length ranging from 0 to 35 nucleotides at a given time. We wanted to test if varying the excess ssDNA length alters the RecA assembly kinetics in the presence of SSB. The passive-nucleation-only model predicts that RecA nucleation time should be inversely proportional to the length of the excess ssDNA^2,12^. In the absence of SSB, the passive nucleation model works for both wtRecA (Figure S3A,B) and RecA E38K (Figure S3C,D), and nucleation time can be fitted by a simple model of ***τ*** ^**−*1***^ = ***k*** **(** ***L*** − ***L***_***o***_ **)** + ***C***, where *τ* is RecA nucleation time, *k* microscopic nucleation rate constant, *L* the total ssDNA length, *L*_0_ the RecA nucleation size (16.8 nt in the fit) and *C* is a constant.

We examined nucleation of RecA E38K in the presence of SSB. In this series of gapped DNA substrates of (dT)_60_-(dT)_100_ and salt concentration used, there is at most one SSB tetramer bound (in (SSB)_65_ binding mode). As the passive-nucleation-only model predicts a sharp decrease in nucleation time as ssDNA gap size increases (shown by the dotted curve in Fig. 2A), surprisingly, we did not observe an apparent nucleation time dependence over these DNA substrates in the presence of SSB (Fig. 2A). These observed nucleation times for RecA assembling onto SSB-ssDNA complex are all in the range ~400–500 seconds (blue filled circles, Fig. 2A), at least 4 times longer than those observed in the absence of SSB (gray open squares, Fig. 2A), confirming the presence of SSB in the prepared SSB-ssDNA complex. In addition, slower extension times were observed in the presence of SSB (Fig. 2B, blue filled circles), about 2-fold slower than those of the SSB-free substrates, again confirming the presence of SSB in the prepared SSB-ssDNA complex. No apparent correlation of the extension times and the excess ssDNA length in the presence of SSB (blue filled circles, Fig. 2B) is observed. The DNA substrates used here included a long (dT)_n_ ssDNA 3′-terminating tail annealed with a short complementary biotin oligo for imaging purpose. Since RecA has been shown to extend predominately from 5′-to-3′^2^, RecA is expected to eventually form filament over the whole DNA substrate except the 351 bp duplex region. This was indeed observed, since the bead BM change varied linearly with the DNA length as expected (Fig. 2C), reflecting that RecA eventually displaces SSB and extend to the full length of ssDNA substrates, with continuation onto the short 19 bp dsDNA region attached to the bead.

Because the longer nucleation times and slower extension times, as well as no apparent ssDNA length-dependence, were seen in these SSB-wrapped DNA substrates ((dT)_n_, n = 60–100), we conclude that either little or no free ssDNA was presented to RecA for nucleation to occur, or interaction between RecA and SSB took place. At lower RecA E38K concentration (0.18 μM), nucleation times of E38K assembly onto SSB-wrapped DNA substrates ((dT)_n_, n = 60, 70, 80 and 90) all become slower but still independent of ssDNA lengths (blue open circles, Fig. 2A), excluding the possibility that observed nucleation process is diffusion-limited. SSB has been shown to slide over ssDNA on a millisecond time scale^24^. What if this fast sliding of SSB covers all ssDNA during the RecA nucleation time scale, so no free ssDNA length can be seen by RecA? If that is the case, based on the passive-nucleation-only model, it is then impossible for RecA nucleation event to take place at all, contrasting with the assembly events we observed on the time scale of 400–500 sec. Next, if an SSB tetramer slides over the whole ssDNA region (60–100) on a slower time scale, the probability of SSB covering a given ssDNA length at a given time would still be different for these (dT)_60–100_ substrates. In other words, different SSB patrolling frequencies over these ssDNA lengths would still lead to different nucleation times. Therefore, the absence of an apparent ssDNA length dependence of nucleation times indicates that an additional nucleation mechanism must exist. Based on the absence of a ssDNA length dependence (Fig. 2A), and the absence of RecA assembly on dsDNA on our 20-minute time scale (Figure S2), we propose that RecA likely interacts with the SSB-ssDNA complex for a nucleation event to take place.

### The RecA E38K nucleation time depends on numbers of SSB

**Figure 3:**
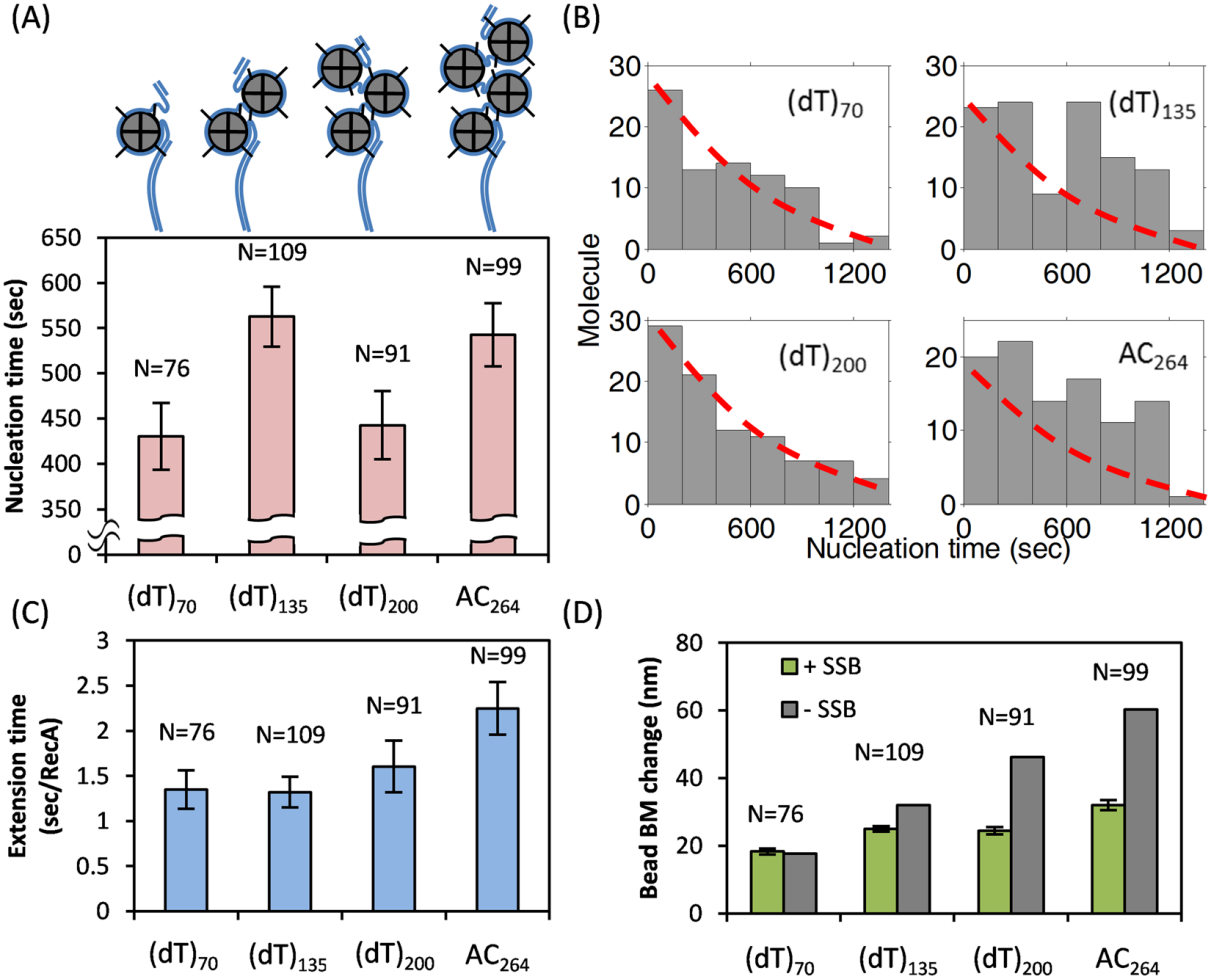
RecA E38K filament assembly dynamics vary with numbers of SSB wrapped on ssDNA. Mean nucleation time (**A**), histograms of nucleation time (**B**), mean extension time (**C**) and mean bead BM change (**D**) of RecA filament assembly on (dT)_70_, (dT)_135_, (dT)_200_ and AC264 gapped DNA substrates. The reaction condition used results in SSB in (SSB)_65_ binding mode, leading to 1–4 SSB bound in these substrates. Error bar is one standard error of the mean. Red lines in (**B**) are exponential fits.

If a RecA interaction with SSB-ssDNA is involved in RecA nucleoprotein filament formation, varying the SSB numbers in the SSB-ssDNA complex should alter the RecA filament assembly kinetics. To test this hypothesis, we prepared 4 gapped ssDNA substrates ((dT)_70_, (dT)_135_, (dT)_200_ and AC264), capable of loading up to 1, 2, 3 and 4 SSB tetramers in the (SSB)_65_ binding mode, respectively (Fig. 3A). Electrophoretic mobility shift assay (EMSA) data showed that each of these SSB-ssDNA complex exist predominantly as a single, high-molecular-weight species in our experimental condition, reflecting the DNA substrates likely bound with the maximum numbers of SSB predominately as designed (Figure S4), although a mixture of SSB binding numbers could still exist. When we challenged these SSB-ssDNA complexes with RecA E38K, we found that nucleation times of RecA E38K filament indeed varied with SSB numbers of SSB-ssDNA complex, but not in a simple pattern (Fig. 3A). Interestingly, (dT)_70_ (accommodating at most 1 (SSB)_65_) and (dT)_200_ (up to 3 (SSB)_65_) substrates showed similar and relatively faster nucleation times, while (dT)_135_ (up to 2 (SSB)_65_) and AC264 (up to 4 (SSB)_65_) substrates produced a slower nucleation time. Pairwise t-test analysis suggested a significant statistical difference between the RecA nucleation times in the odd SSB bound numbers and those in the even SSB bound numbers substrates (Table S1). In addition, nucleation time histograms of (dT)_70_ and (dT)_200_ distribute differently from those of (dT)_135_ and AC264 (Fig. 3B). For example, histograms in odd-numbered SSB substrates show a rather exponential-like decrease, but the even-numbered SSB substrates show an additional peak around 600–1000 seconds. The extension times of RecA assembly on these SSB-wrapped ssDNA complex are rather similar, with a slightly longer time required for increasing numbers of SSB bound (~1.35–1.60 sec/RecA for (dT)_70_, (dT)_135_ and (dT)_200_, and 2.24 sec/RecA for AC264 substrates, Fig. 3C). These numbers are on the similar time scale of the extension time observed in Figure 2B (~1.5 sec/RecA). This indicates that RecA encounters a similar barrier during the extension process, perhaps reflecting SSB migration along the ssDNA ahead of the RecA filament extension. The slightly longer extension time observed in AC264 substrates might reflect that RecA addition requires more activation energy to overcome stronger SSB-ssDNA interactions in AC264 substrates. On the other hand, we observed a smaller bead BM change on (dT)_135_, (dT)_200_ and AC264, meaning RecA may not be capable of displacing all SSB when there are multiple SSB bound to the ssDNA substrates (Fig. 3D). The apparent difference in RecA nucleation times among different SSB numbers confirms our hypothesis that interactions between RecA and SSB-ssDNA exist for RecA assembly on the SSB-wrapped DNA substrates. The changes suggest that adjacent SSB tetramers may align head to tail on ssDNA, with a suitable surface for RecA nucleation presented at every other tetramer.

### SSB C-terminus truncation significantly reduces RecA filament assembly

**Figure 4:**
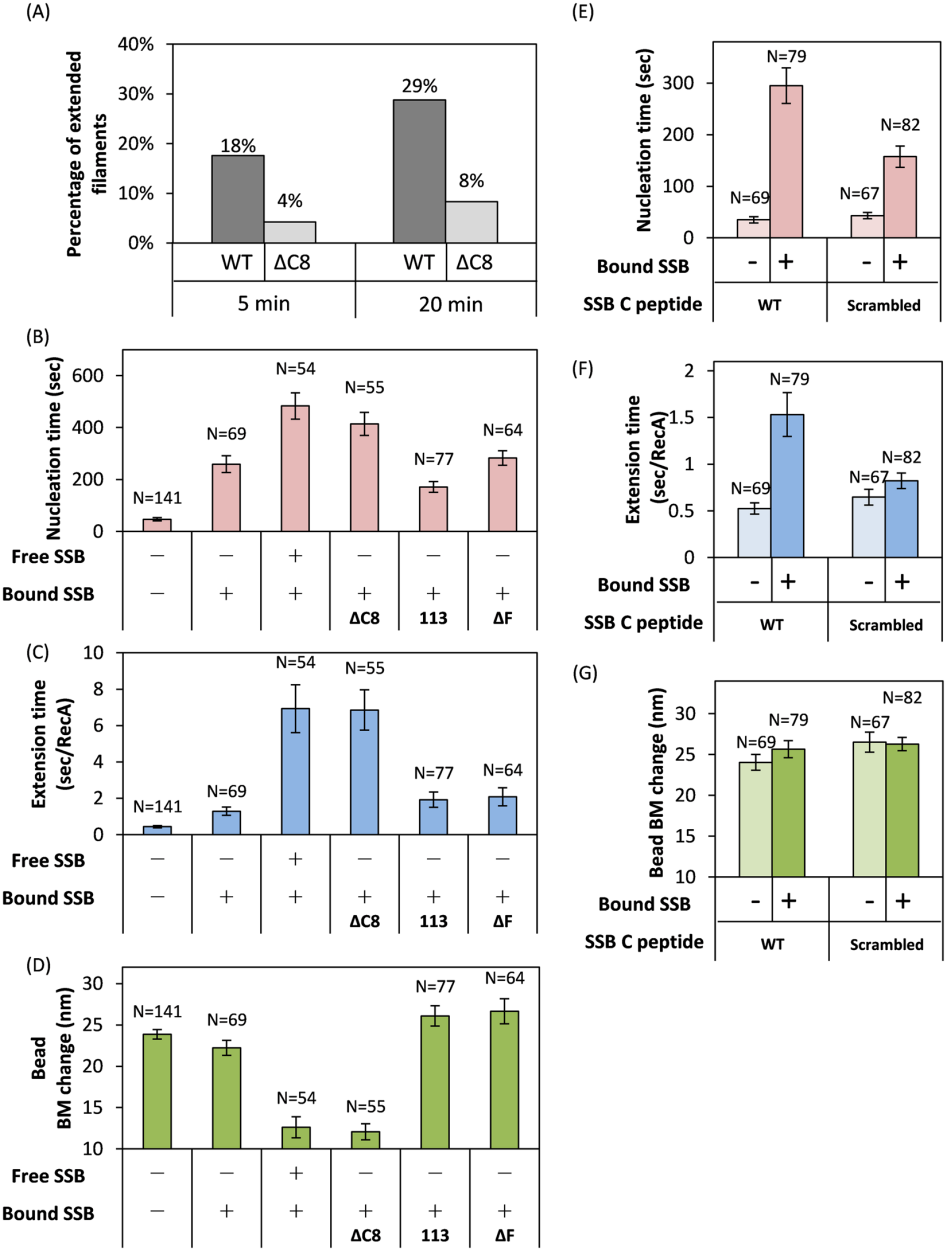
Modifications to SSB alter RecA E38K filament assembly dynamics. (**A**) Percentage of the extended RecA E38K filament in the presence of bound wtSSB (WT) or bound SSBΔC8 after 5 minutes and 20 minutes of RecA E38K addition (see Figure S5). (**B**–**D**) Mean nucleation time (**B**), mean extension time (**C**) and bead BM change (**D**) of RecA E38K on ssDNA substrates associated with various SSB proteins and mutants. (**E**–**G**) Mean nucleation time (**E**), mean extension time (**F**) and bead BM change (**G**) of RecA E38K incubated with 2 μM SSB C-terminal peptides (wild-type or scrambled). The E38K-C peptide mixture was flowed into the reaction chamber containing the SSB-wrapped ssDNA substrates. Pre-incubation with the wild-type C-peptide of SSB significantly delays nucleation and extension of RecA E38K, compared to the experiments using scrambled C-peptide of SSB. All assembly kinetics were determined using (dT)_90_ gapped ssDNA substrates, associated with either no SSB, bound wtSSB, bound wtSSB plus free wtSSB, bound SSBΔC8, bound SSB113 or with bound SSBΔF. Error bar is one standard error of the mean.

*E*. *coli* SSB is known to interact with many DNA enzymes through its C-terminus^16,23^. We hypothesize that RecA likely interacts with SSB through its C-terminal region. However, in the absence of ssDNA, the C-terminal region of SSB is mostly buried^35^, so no detectable RecA-SSB interaction was observed in the absence of ssDNA^30^. If there exists an interaction of RecA-SSB through the C-terminal domain of SSB, we anticipate that weakened RecA-SSB interaction would significantly lower the RecA assembly efficiency in the SSB C-terminus mutants. To test this hypothesis, we used a C-terminal truncation SSB mutant (SSBΔC8) that did not compromise the SSB tetramer structure^35^. This SSB truncation mutant did not affect ssDNA affinity significantly, as seen in both gel electrophoretic mobility shift assay (EMSA) and surface plasmon resonance (SPR) experiments under several buffer conditions and ssDNA lengths (Figure S7 and Table 1). We compared how RecA E38K displaces wtSSB and SSBΔC8 on (dT)_90_ gapped DNA in real-time, but very little assembly product was found for SSBΔC8 within our reaction time (up to 20 minutes, Fig. 4A). A significantly reduced RecA filament assembly observed for SSBΔC8 mutants provides additional evidence for the presence of RecA-SSB interaction and may indicate that the interaction domain is located within the C-terminus of SSB.

**Table 1:** Summary of ssDNA binding affinity of wild-type SSB and SSBΔC8 determined from EMSA and SPR experiments.

Method	buffer	ssDNA length (nt)	K_d_ (M)		Figure S7
			wtSSB	SSBΔC8	
EMSA	1	55	4.04 × 10^−9^ (1)	1.39 × 10^−8^ (~3.4)	(A)
	1	75	1.14 × 10^−9^ (1)	7.52 × 10^−9^ (~6.6)	(B)
SPR	1	55	2.40 × 10^−8^ (1)	4.70 × 10^−9^ (~0.2)	(C)
	2	55	1.45 × 10^−7^ (1)	8.91 × 10^−8^ (0.6)	(D)

*Numbers in the parentheses refer to the relative change in fold.

### Different SSB binding modes affect RecA assembly

With much-reduced assembly efficiency, a very small fraction of RecA filament assembly events was observed on SSBΔC8-wrapped DNA substrates. These events, however, were kinetically distinct from those in wtSSB-wrapped DNA substrates (Fig. 4B–D, column 4). Under the reaction condition that SSB are bound onto ssDNA and no free SSB is available, RecA nucleation time increased by ~150% (~259 sec for wtSSB, second column in Fig. 4B, to ~414 sec for SSBΔC8, fourth column) and extension time by ~540% (~1.28 sec to ~6.86 sec, Fig. 4C). In addition, a reduced bead BM change in SSBΔC8 suggested incomplete removal of SSB (Fig. 4D). A previous single-molecule FRET study suggested that truncation of the C-terminal 42 amino acids of wtSSB leads to (SSB)_35_ binding mode preference^17^, suggesting that deletion of SSB likely might lead to different binding modes. In our experimental salt condition (10 mM Mg^2+^), wtSSB was likely in the (SSB)_65_ binding mode. The relatively fast nucleation time in wtSSB (~259 sec) likely reflects the RecA assembly against the (SSB)_65_ binding mode, while the slower nucleation time against SSBΔC8 (~414 sec) likely reflects that RecA assembly against the SSB binding mode different from the (SSB)_65_. Therefore, the dramatic differences in RecA assembly against wtSSB and SSBΔC8-wrapped substrates suggest that (i) different SSB wrapping modes modulate the RecA-SSB interaction, and (ii) RecA filament assembly involves a RecA-SSB interaction.

In order to confirm that different SSB binding modes indeed modulate RecA-SSB interaction, in turn regulating RecA assembly, we monitored RecA assembly kinetics in DNA substrates wrapped by other well-documented, different SSB binding modes. A previous study showed that just by including excess SSB in solution triggers the SSB binding mode conversion between (SSB)_65_ and (SSB)_35_^17^. All our earlier experiments included only SSB bound to DNA substrates, accompanied by extensive buffer wash to remove any free SSB in the solution before introducing RecA. The (SSB)_65_ mode was based on the salt condition used. In the new experiments, additional free SSB molecules are included at the time at which RecA was introduced into the reaction chamber. Thus, excess SSB exists in the solution to allow SSB binding mode conversion between (SSB)_65_ and (SSB)_35_. Including excess SSB produces a significant change in RecA assembly kinetics (Fig. 4B–D, column 3). Dramatic increases in nucleation time and extension time were observed, and a reduction in bead BM change confirmed that SSB binding mode alters RecA assembly kinetics, likely by the model that different SSB binding modes alter RecA-SSB interaction. This is also confirmed by the data in bead BM change (Figure S6). Knowing that excess SSB can induce conversion into the (SSB)_35_ binding mode (Fig. 4B–D, column 3), and its striking similarities to that in SSBΔC8 (bound only, Fig. 4B–D, column 4), we speculate that SSBΔC8 likely could adopt the (SSB)_35_ binding mode in our experimental condition. Previous single-molecule magnetic tweezers study showed that RecA binding on ssDNA was outcompeted by SSB at higher concentration (>100 nM)^36^. However, here we only included 50 nM SSB tetramer to the solution, the possibility that SSB outcompeted RecA from ssDNA was excluded.

We also studied the RecA E38K assembly on two other SSB point mutants, SSB113 and SSBΔF that were previously shown to have a defect in interacting with other proteins^37,38^. As seen in Fig. 4B–D (column 5–6), RecA assembly kinetics showed no apparent difference for these two SSB point mutants and wtSSB. This suggests that multiple RecA-SSB interactions likely exist, and removing just one contact is not significant in disrupting the RecA-SSB interactions. To further confirm the RecA-SSB interactions, we masked the RecA domains that interact with SSB using a C-terminal peptide of SSB. The idea is that if the RecA-SSB interactions exist and are important for RecA filament assembly, masking this interaction domain by the interaction-mimic C-terminal SSB peptides (C peptides) will abolish this assembly pathway and retard the assembly process. To do this, we first mixed RecA E38K and an equal molar amount of SSB C peptides (2 μM) in the solution for 20–25 min and then monitored the kinetics of this RecA-peptide mixture on the SSB-wrapped ssDNA substrates. Compared to the control experiments using scrambled peptides of the same length, wild-type C peptides indeed show longer nucleation time (Fig. 4E) and extension time (Fig. 4F). Both wild-type and scrambled C peptides place no effects on final RecA filament length, as seen in 4D (column 1–2) and Fig. 4G. Both nucleation and extension time of RecA E38K assembly on SSB-(dT)_90_ substrate were diminished as an equal molar amount of scrambled SSB C peptides were added (column 2 in Fig. 4B; column 4 in Fig. 4E). Furthermore, adding more (20 μM) peptides significantly alter the RecA assembly, likely because μM-amount of negatively-charged peptides created a local crowding effect, as shown previously^15^. Nonetheless, we peculiarly focus on the difference in RecA E38K nucleation and extension kinetics between wild-type peptide experiements and scrambled peptide ones. These combined observations further support that RecA-SSB interactions exist and play a role in RecA filament formation.

## Discussion

The central role of RecA in bacterial recombination requires its assembly onto ssDNA to form nucleoprotein filaments, the first commitment step subject to tight regulation^3,8^. *In vivo*, ssDNA is likely bound by other proteins, most commonly by SSB in *E*. *coli*. SSB-ssDNA complex formation is useful not only for preventing nucleolytic degradation of ssDNA but also for removing potential secondary DNA structures. However, this SSB-ssDNA complex prevents RecA from direct access to ssDNA, leading to the slow kinetics and complexity of RecA filament assembly on SSB-wrapped DNA. On the other hand, inclusion of SSB stimulates RecA D-loop and strand exchange reactions *in vitro* ^39–42^, likely by SSB removing secondary structure of ssDNA to facilitate RecA filament extension^24^. Therefore, the SSB role in RecA-mediated recombination process is multi-faceted.

Here, we studied how RecA interacts with SSB-wrapped DNA, using various ssDNA lengths and SSB numbers, during the RecA filament assembly. Previous studies on how RecA assembles on SSB-wrapped ssDNA have provided information towards a molecular model^2,12,24,28,30,43–45^. The more generally accepted model includes RecA-ssDNA and SSB-ssDNA interactions and points to RecA nucleation on free ssDNA not covered by SSB – namely the passive-nucleation-only model. The DNA interaction with RecA and SSB are mutually exclusive, and pull-down experiments in the absence of ssDNA did not capture significant RecA-SSB interaction^30^. However, the absence of an apparent ssDNA-length dependence for RecA nucleation observed over the ssDNA length of 60–100 nt with bound SSB in the (SSB)_65_ binding mode (Fig. 2) calls for other RecA nucleation pathway(s) in addition to the passive-nucleation-only model. Even though that SSB in a (SSB)_65_ binding mode could slide, and likely shield ssDNA more than 65 nt^21,23,24^, the SSB patrolling frequency must decrease with increasing ssDNA length, and the SSB shielding is less effective over longer ssDNA. It is also possible that SSB sliding occurs on an extremely fast time scale over the ssDNA lengths, so the whole ssDNA length is effectively shielded. However, this scenario will completely abolish RecA-ssDNA interaction and lead to no filament assembly based on a passive-nucleation-only model. It is possible that either the intrinsic disordered region or the acidic tip of SSB alters the SSB binding modes, cooperativity or dynamics on DNA^25^. However, these alterations in SSB cannot account for the nearly similar RecA nucleation times seen over the wide range of ssDNA lengths (Fig. 2A). Thus, some RecA-SSB interaction must exist in addition to the RecA-ssDNA interaction.

Our observation that the RecA nucleation time depends on the bound SSB numbers also suggests an interaction between RecA and ssDNA-bound SSB (Fig. 3). This RecA-SSB-ssDNA interaction likely could be weak or transient, as it is not detected in the pull-down assay^30^. Many DNA-binding proteins interact with the highly conserved C-terminal region of SSB^16^. The SSB C-terminus is highly acidic, resembling the negatively charged ssDNA backbone. It has been suggested that SSB acts as DNA maintenance hub by using its ssDNA-like C-terminus fragment to interact with other DNA enzymes, as has been implicated in regulating the assembly of replisome and of RecA filaments^46^. The SSB C-terminal truncation mutant, SSBΔC8, produces a much lower RecA assembly efficiency (Fig. 4), confirming the RecA-SSB interaction suggested. The C-terminal region of SSB has been found to interact with the core domain of SSB tetramers and thus not exposed in the absence of ssDNA. However, this C-terminal tail and core domain interaction of SSB diminishes when SSB binds to ssDNA^35^. It is possible that the C-terminal region of SSB is not available to interact with RecA in the absence of ssDNA, as seen in the pull-down assay^30^. Furthermore, this C-terminal region of SSB has been suggested to be critical in modulating the binding mode conversion of (SSB)_65_ and (SSB)_35_^25^. Considering our observation that (SSB)_65_ and (SSB)_35_ binding mode conversion significantly affects RecA nucleation (Fig. 4), it is possible that the binding of the C-terminus of SSB to RecA modulates the SSB binding modes, its conversion, and thus the RecA assembly process.

wtRecA showed significantly reduced DNA-dependent ATPase activity when SSB is present, reflecting the reduction in RecA filament assembly when SSB was bound to ssDNA. It was shown that RecA E38K, a C-terminus deletion (RecAΔC17) or double mutants (RecA E38K/ΔC17) all have enhanced capacity to bind to SSB-coated ssDNA, compared to wtRecA, as measured indirectly using the DNA-dependent ATPase activity^27,31,32^. Our results here directly demonstrated the enhanced SSB-displacement activity of E38K. An even faster SSB-displacement activity of E38K/ΔC17 double mutants was observed^27^. It is likely that these mutants efficiently unmask part of the RecA domain that is responsible for interacting with SSB. It has been shown that the RecOR complex stimulates RecA loading on SSB-ssDNA complex^2^. All existing models propose a direct RecOR-SSB interaction responsible for this stimulation^47,48^. Knowing that RecA-SSB interaction exists, it is also possible that the RecOR stimulation could take place by unmasking a RecA domain needed to facilitate RecA-SSB interactions.

Once RecA successfully nucleates on SSB-wrapped ssDNA, it starts to extend by the association of more RecA proteins. We found longer RecA extension time (time required to add a RecA molecule, sec/RecA) is required in the presence of SSB (Fig. 1), implying that RecA encounter barriers during extension, likely reflecting to push away SSB. Interestingly, we found both the SSBΔC8 mutant or free wtSSB significantly increased RecA extension time (Fig. 4C). This suggests that (SSB)_65_ and (SSB)_35_ binding modes resist RecA extension differently. The (SSB)_35_ wrapping on ssDNA has been suggested to be more static, in contrast to the highly dynamic nature of the (SSB)_65_ binding mode^21,23,24^. Looser interaction of SSB-ssDNA in the (SSB)_65_ binding mode could allow faster RecA extension. When RecA filament assembly process displaces SSB from the ssDNA-substrates, free SSB or other SSB-interacting proteins could modulate the SSB binding mode into a harder-to-be-displaced binding mode, such as (SSB)_35_ one, and thus retard RecA filament extension. If RecA molecules become limited during extensive DNA damage *in vivo*, such a change in RecA extension time resulting from SSB binding mode conversion could serve as a RecA budgeting mechanism for proper and efficient repair.

In our TPM experiments, bead BM change reflects the assembled length of the RecA filament. Since an excess amount of RecA E38K was used, maximum RecA coverage is expected, even though the ATP hydrolysis could lead to some filament dynamics due to the disassembly of RecA. We indeed observed nearly full RecA filament assembly in the absence of SSB. In the presence of single SSB tetramer in (SSB)_65_ binding mode, we also observed nearly full RecA coverage (Fig. 2C). The similar bead BM changes with and without SSB tetramer in (SSB)_65_ binding mode indicates the complete removal of SSB with RecA filament assembly (Fig. 4D). However, in the presence of multiple SSB tetramers on ssDNA, SSBΔC8, or with free SSB, a reduction in observed bead BM change was seen (Figs 3D and 4D). The inability of RecA to completely remove SSB in these conditions likely reflects the different SSB binding modes present. Consistent with this, the histogram of bead BM change of (dT)_90_ + SSB in the presence of free SSB showed two populations (Figure S6). The larger bead BM change corresponds to nearly full RecA filament formation (Figure S6). The smaller bead BM change peak (~6.7 nm) reflects a shorter RecA filament, with the difference likely corresponding to a size of two SSB tetramers in (SSB)_35_ binding mode remaining on ssDNA. Such a continuous association of SSB at the end of a RecA filament was suggested previously^44^. We noted that this phenomenon is unlikely a consequence of the slower extension time in the presence of free SSB (Fig. 4C) because all single-molecule time-courses reached a steady plateau by the end of the recording. SSBΔC8 experiments also showed a similar bead BM change pattern (Figure S6).

**Figure 5:**
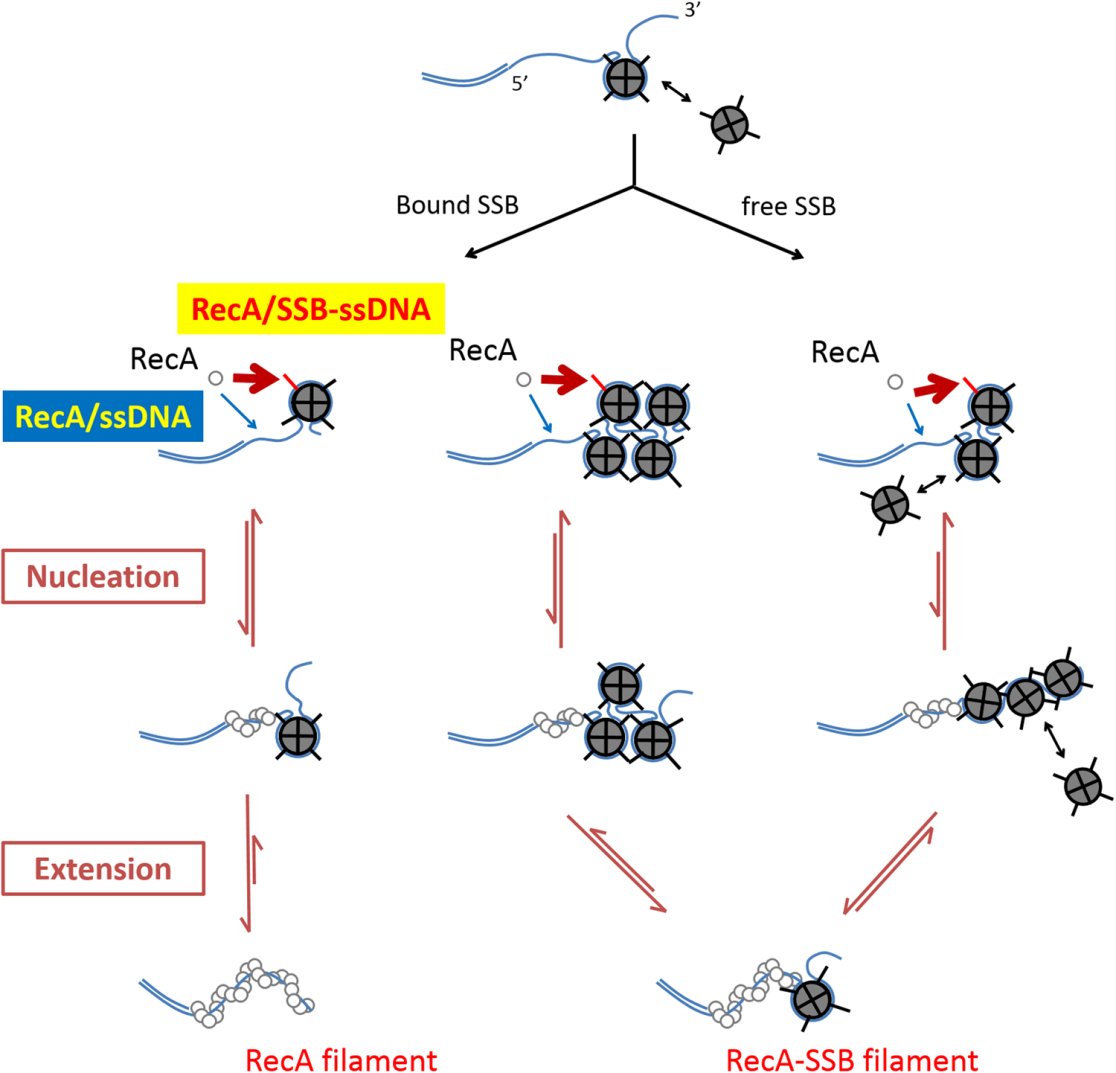
A model of RecA filament assembly on SSB-wrapped ssDNA substrates. In the beginning, SSB wraps around ssDNA tail with high affinity (Top). In case there is only singly bound SSB in its (SSB)_65_ binding mode (left panel), RecA has to either interact with the C-terminus of SSB or directly assemble on exposed ssDNA region to form a stable nucleus, followed by fast extension and complete removal of SSB. Once ssDNA is bound by multiple numbers of SSB (middle panel), RecA spends longer time nucleating and extending on ssDNA, especially for even-numbered SSB DNA substrate. RecA is incapable of displacing entire SSB on ssDNA. In the presence of excess SSB (right panel), SSB prefers (SSB)_35_ binding mode; the highly cooperative nature of (SSB)_35_ binding mode forces RecA to nucleate in a slower mechanism, followed by slower extension and incomplete removal of SSB.

In addition to the previously identified passive RecA nucleation, our results demonstrate the existence of a RecA-SSB interaction through the SSB C-terminus (Fig. 5). RecA nucleation can occur on free, exposed ssDNA when available. However, when a segment of exposed ssDNA is not accessible, RecA can interact directly with SSB-ssDNA complex to initiate the assembly process. The RecA-SSB interaction and RecA assembly depends on SSB binding modes. The RecA nucleation event likely occurs faster for the (SSB)_65_ binding mode than for the (SSB)_35_ binding mode. After a RecA nucleation event takes place, RecA filament extension involves pushing SSB away (Fig. 5). The SSB binding mode and its conversion affect multiple steps in RecA filament assembly, as in nucleation, extension and filament length. It, therefore, suggests a SSB regulation mechanism on RecA filament assembly. This RecA-SSB interaction, however, cannot be stable and long-lasting, as a stable interaction would then have SSB stay bound to ssDNA, making RecA filament formation inefficient. Many details of this RecA-SSB interaction remain to be explored: How does RecOR facilitate RecA filament assembly? How does the crowded environment present *in vivo* affect the RecA-SSB interaction^15,49,50^? Experiments with higher spatiotemporal resolution and ability to monitor SSB and RecA simultaneously are useful to define these molecular events.

## Conclusion

We monitored RecA E38K filament formation on SSB-wrapped ssDNA in real-time at the single-molecule level. In addition to nucleation on free ssDNA, we showed that there exists an additional pathway that RecA filaments assemble through a RecA-SSB interaction. This pathway is apparently slower in kinetics but takes place when no other accessory proteins are available. We showed that the SSB binding modes and conversion among these binding modes are involved in regulating the RecA assembly process. When limited RecA proteins are available, SSB proteins could function as a DNA maintenance hub to budget RecA for efficient recombinational repair events.

## Materials and Methods

### Protein and buffer condition

*E. coli* wild-type RecA and RecA E38K mutant were purified as previously described^27^. *E*. *coli* wild-type SSB protein was purchased from Promega. SSBΔC8 was purified as previously described^35^. SSB 113 and ΔF mutants are kind gifts from Jim Keck (Wisconsin). RecA assembly reactions were carried out in the presence of 25 mM Tris-HCl, 10 mM magnesium acetate, 3 mM potassium glutamate at pH 7.5 (buffer 1). To study the SSB-ssDNA binding affinity, reactions were done in 10 mM Tris, 0.1 mM Na3EDTA, pH 8.1 (buffer 2) for comparison purpose^35^. All single-molecule experiments included additional 1 mg/mL BSA to prevent non-specific surface interactions. All SPR reactions included 0.005% tween-20. The SSB C-terminal peptide WMDFDDDIPF, and a sequence-scrambled control peptide WDFMDDPFID was synthesized by and purchased from the Biotechnology Center at the University of Wisconsin-Madison.

### Synthesis of gapped ssDNA substrates

AC264 gapped ssDNA substrate consists of 349 bp long dsDNA handle, a 264 nt ssDNA gap containing predominantly alternative AC sequence and a 24 bp short dsDNA handle, synthesized as previously described^51^. (dT)_n_ gapped DNA consists of a 351 bp long dsDNA handle, a ssDNA gap containing (dT)_n_ (n = 35–200 nt) and a 19 bp short dsDNA handle. A 351 bp dsDNA was first prepared by a PCR reaction using a digoxigenin-capped primer (5′-digoxigenin-TGCCACCTTTTCAGCTCG-3′), a regular primer (5′-ATCGGTCGACGCTCTCCCTT-3′), and pBR322 as a template. A nick on this 351 bp dsDNA was generated using Nt. BstNBI nicking enzyme (NEB), then challenged by an excess of the regular primer to generate 331/351 hybrid. Oligos with various length of (dT)_n_ capped by sequence complementary to the sticky end of the 331/351 hybrid and a biotin-capped primer (5′-biotin-CGGATGGCATGACAGTAAG-3′) were pre-treated with T4 PNK (NEB), followed by ligation with the 331/351 hybrid in the presence of T4 DNA ligase (NEB). All DNA substrates were purified by gel extraction. DNA primers were purchased from Gene Link (primer containing (dT)_200_), Integrated DNA Technologies (primers containing (dT)_35_, (dT)_60_, (dT)_65_, (dT)_70_, (dT)_72_, (dT)_75_, (dT)_78_, (dT)_80_, (dT)_90_, (dT)_100_ and (dT)_135_) and Bio Basic Inc. (the digoxigenin-capped, biotin-capped and regular primers).

### Single-molecule assembly experiment

The reaction chamber was prepared as previously described^9^. Gapped ssDNA substrates were added in 4 nM to the anti-digoxigenin-coated coverslip, followed by the annealing of an excess of the biotin-capped primer on-chip. Streptavidin-labeled polystyrene beads (220 nm, Bangs Laboratories Inc.) were then attached to the DNA substrates for single-molecule imaging purpose. Single-stranded DNA binding protein (SSB), either wtSSB, SSBΔC8, SSB113 or SSBΔF was added in the tetramer concentration of 50 nM at room temperature for 15 min. Unbound SSB was eliminated by an extensive buffer wash. Reaction buffer containing 2 μM RecA mixed with 1 mM DTT and 2 mM ATP was pre-incubated at 37 °C for 10 min off-line. This RecA mixture was added to the reaction chamber containing bead-labeled gapped DNA substrates after cooled down to room temperature. Images were taken at 33 ms resolution using Dage MTI camera, as previously described^9^. Bead centroid position was fitted to 2-D Gaussian through Glimpse and corrected for stage drift by a pre-adsorbed stuck bead^9^. Bead BM amplitude was defined as standard deviation of 20 images using sliding window. For time-lapse experiments, image acquisition was initiated about 30 seconds (1000 image frames) before addition of RecA mixture and was terminated about 20 minutes (40000 image frames) after addition of RecA mixture. For snapshot experiments, 1000 image frames were recorded at 7–8 different positions on coverslip beginning at the time points specified. In snapshot experiments, tethers with bead BM greater than 55 nm in (dT)_90_ gapped substrate were qualified as extended RecA filament (Figures S1B,C,H,I and S5). To ensure single molecule measurements, low anti-digoxigenin concentration (5 μg/mL) was used to sufficiently reduce the surface DNA density. Also, only beads with similar Brownian motion amplitudes in x- and y-axis were analyzed.

## Electronic supplementary material

Supplemental information
