# Supplementary material for: RecA-SSB Interaction Modulates RecA Nucleoprotein Filament Formation on SSB-Wrapped DNA
Source: Sci Rep. 2017 Sep 19;7:11876. doi: 10.1038/s41598-017-12213-w (PMC5605508; doi:10.1038/s41598-017-12213-w)
Supplement: Supplementary file 1 — Supplemental information [file 41598_2017_12213_MOESM1_ESM.pdf]

Supplemental Information for

RecA-SSB Interaction Modulates RecA Nucleoprotein Filament Formation on SSB-Wrapped DNA

Hung-Yi Wu[#], Chih-Hao Lu[#], and Hung-Wen Li[*]

Department of Chemistry, National Taiwan University, Taiwan

[*] To whom correspondence should be addressed: Hung-Wen Li, E-mail hwli@ntu.edu.tw

#H.Y.W. and C.H.L. contributed equally to this work.

EMSA and SPR experiments.

To compare the ssDNA binding affinity of wild-type (wt) SSB and SSBΔC8, electrophoresis mobility shift assay (EMSA) and surface plasmon resonance (SPR) experiments were carried out. In both experiments, DNA substrates were prepared using a 19 nt 5′-end-labeled ssDNA oligo (Cy3 label for EMSA and biotin label for SPR) annealed to another long ssDNA oligo to generate a ssDNA overhang (55 or 75 nt). In EMSA experiments (Figure S7), given concentrations of wtSSB and SSBΔC8 were mixed with Cy3-labeled DNA, and the mixture was subject to 8% PAGE in 1xTAE, 125 Volt for 2.5 hours at 4°C. The image was quantified using GE Typhoon Trio. Cy3 fluorescence intensity was imaged under 532 nm laser excitation. Each set of experiments included at least 3 repeats. Biotin-labeled DNA substrates were anchored on the streptavidin-decorated surface in the SPR (Biacore T200) measurements.

EMSA experiments were also used to show wtSSB binding modes as a function of SSB tetramer concentration at different ssDNA lengths (80 nt, 155 nt and 220 nt, Figure S4). Each poly-dT-containing DNA oligo was first mixed with Cy3-labeled short DNA oligo in 2:1 molar ratio. A sample of each lane was then prepared by mixing hybrid DNA oligo, SSB, RecA reaction buffer and sucrose for loading purpose. The final sample of each lane was 25 μL, containing 2 nM poly-dT-containing DNA oligo, 1 nM Cy3-labeled short DNA oligo, SSB in indicated tetramer concentration in buffer 1 with 8% w/w sucrose and was subjected to native-PAGE analysis (6-8%) in 1X TAE, 125 volts at 4°C.

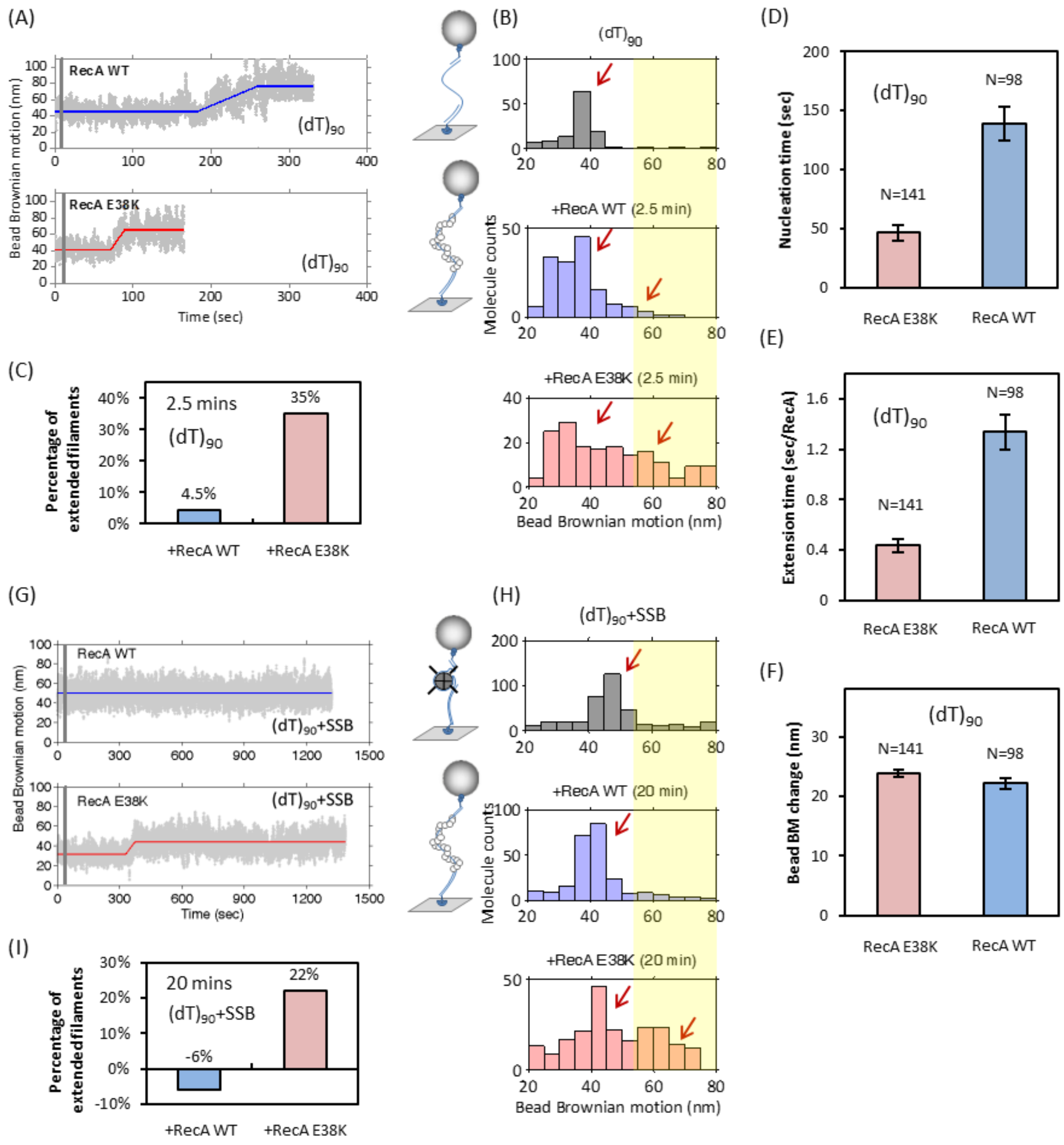

Figure S1. RecA E38K forms filament faster than wtRecA either on bare ssDNA or SSB-wrapped ssDNA. (A) Representative time-courses of wtRecA (top) and RecA E38K (bottom) on bare (dT)90 gapped DNA substrates. (B) Histograms of bead BM of SSB-free DNA substrates (top), and 2.5 minutes after the addition of wtRecA (middle) and RecA E38K (bottom). (C) Quantification of the extended filament percentage after 2.5 minutes of RecA addition (from B) and after 20 minutes of RecA addition. (D) Mean nucleation time, (E) mean extension time and (F) bead BM change of wtRecA and RecA E38K assembly on SSB-free $(dT)_{90}$ substrates. (G) Representative time-courses of wtRecA (top) and RecA E38K (bottom) on single SSB-wrapped $(dT)_{90}$ gapped DNA substrates. The reaction condition used resulted in the $(SSB)_{65}$ binding mode. (H) Histograms of bead BM of SSB-coated DNA

substrates only (top), and 20 minutes after the addition of wtRecA (middle) and RecA E38K (bottom). (I) Quantification of the extended filament percentage after 20 minutes of RecA addition (from H).

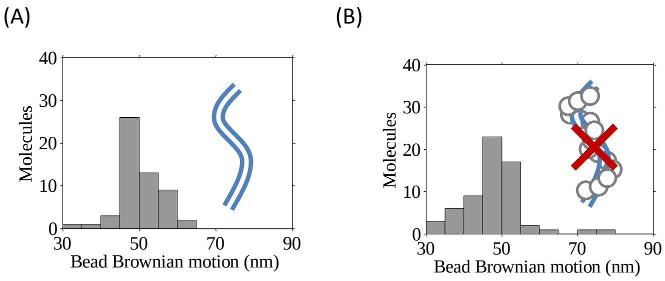

Figure S2. RecA E38K does not assemble on dsDNA under our experimental condition in 20 minutes. (A) Bead BM histograms of fully dsDNA substrates only (439 bp) and (B) in the presence of RecA E38K and ATP. Data were taken after 20 minutes of E38K addition. The dsDNA used in this experiment was obtained through PCR, with a digoxigenin-capped primer (5′-digoxigenin-ACTACGATACGGGAGGGC-3′), a biotin-capped primer (5′-biotin-CGGATGGCATGACAGTAAG-3′) and pBR322 as a template.

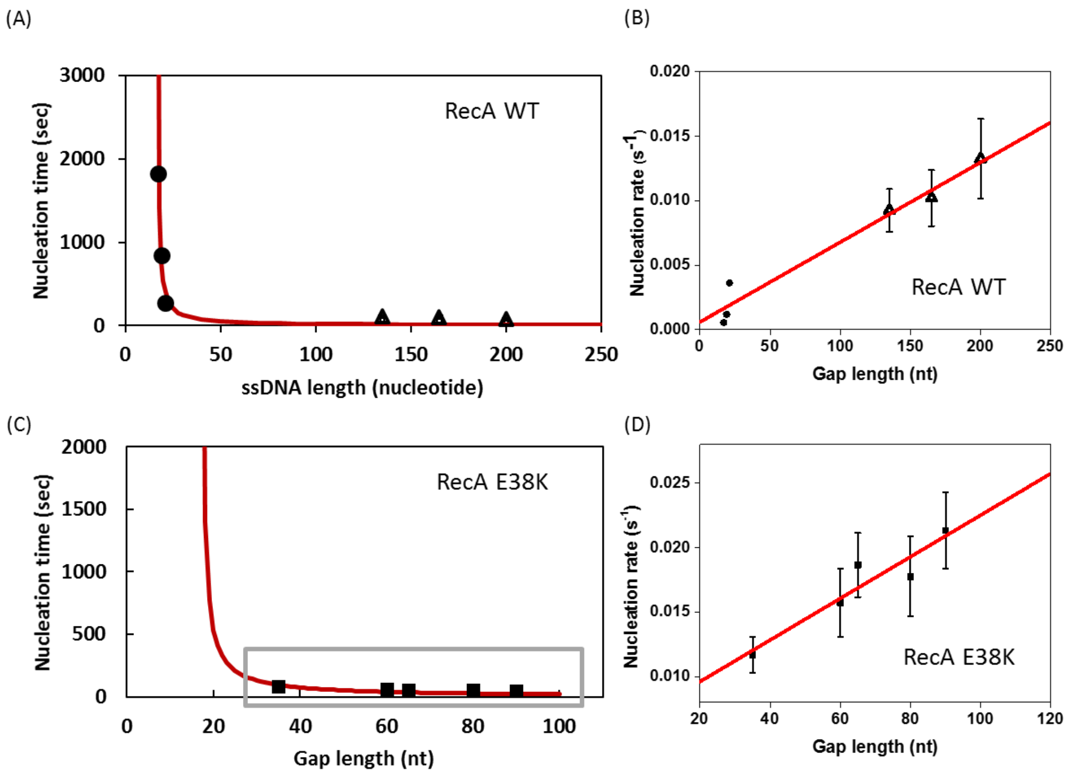

Figure S3. Nucleation times of both (A) wtRecA and (C) RecA E38K are inversely proportional to ssDNA length in the absence of SSB. Black filled circles in (A) & (B) are data from Joo et al. (2006), and open triangle data were done using TPM experiments on gapped DNA substrates of different ssDNA lengths. The red lines in (A) and (C) are fitted by $\tau^{-1} = k(L - L_o) + C$, where τ, k and L are mean nucleation time, microscopic rate constant, ssDNA length, and constant, respectively. L_o is 16.8 nt by fit. Data points in (A) and (C) were drawn as mean nucleation rate against ssDNA length plot shown in (B) and (D), respectively. As expected, with longer ssDNA lengths, the nucleation rates of both wtRecA and RecA E38K increase. There are linear relations between nucleation rate and ssDNA gap length in both wtRecA and RecA E38K assembly. Error bar is one standard error of mean.

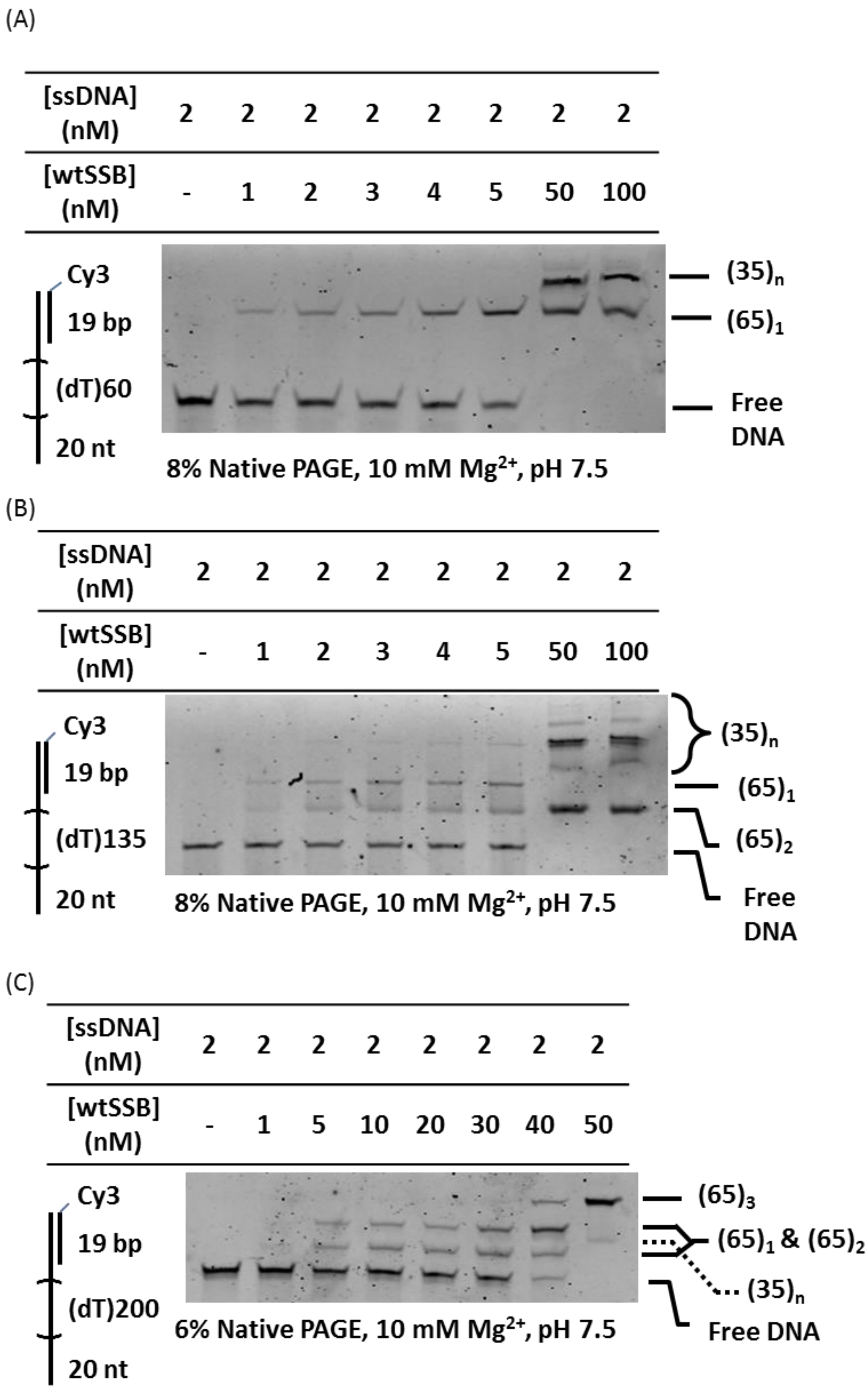

Figure S4. An electrophoretic mobility shift assay (EMSA) was used to identify wtSSB binding mode as a function of SSB tetramer concentration at different ssDNA lengths (A, 80 nt; B, 155 nt; C, 220 nt).

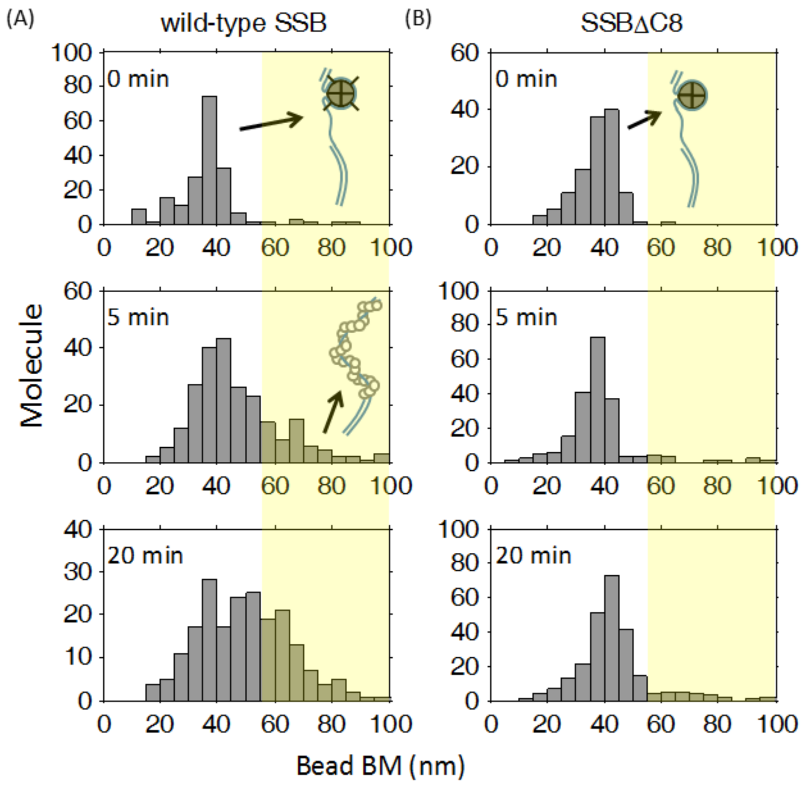

Figure S5. RecA E38K shows different filament assembly efficiencies on the $(dT)_{90}$ gapped DNA substrate wrapped by wild-type SSB (A) and by SSB C-terminus truncation mutant, SSBΔC8 (B).

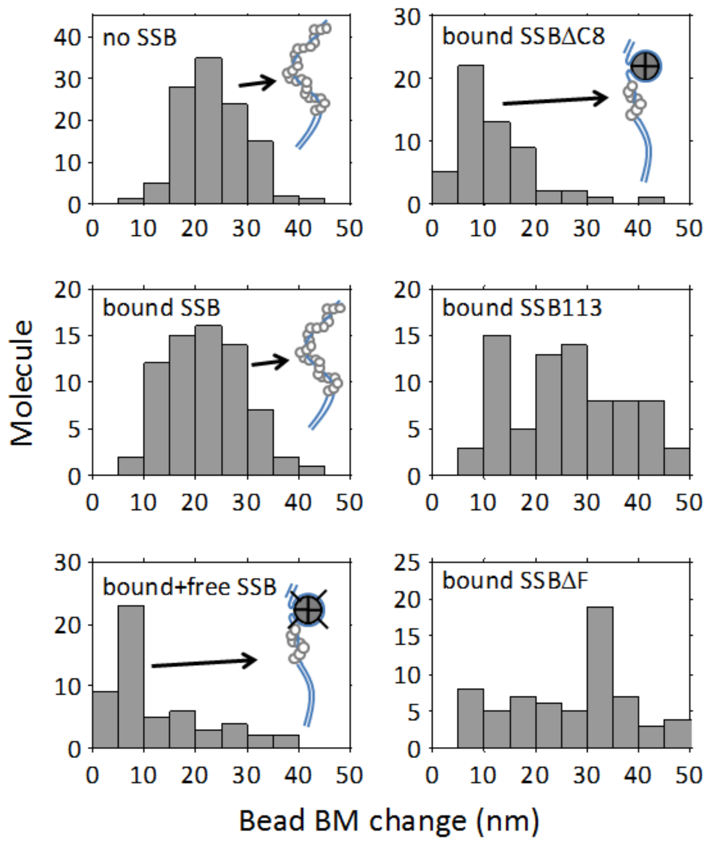

Figure S6. Histograms of bead BM change upon RecA filament assembly from Figure 4D. Assembled RecA filament is indicated by a bead BM change of 22.7±6.8 nm. An additional peak at smaller bead BM change of 6.6±3.0 nm was observed when the reaction included free wtSSB in the solution or when SSBΔC8 mutant was used. Error bar is one standard error of the mean.

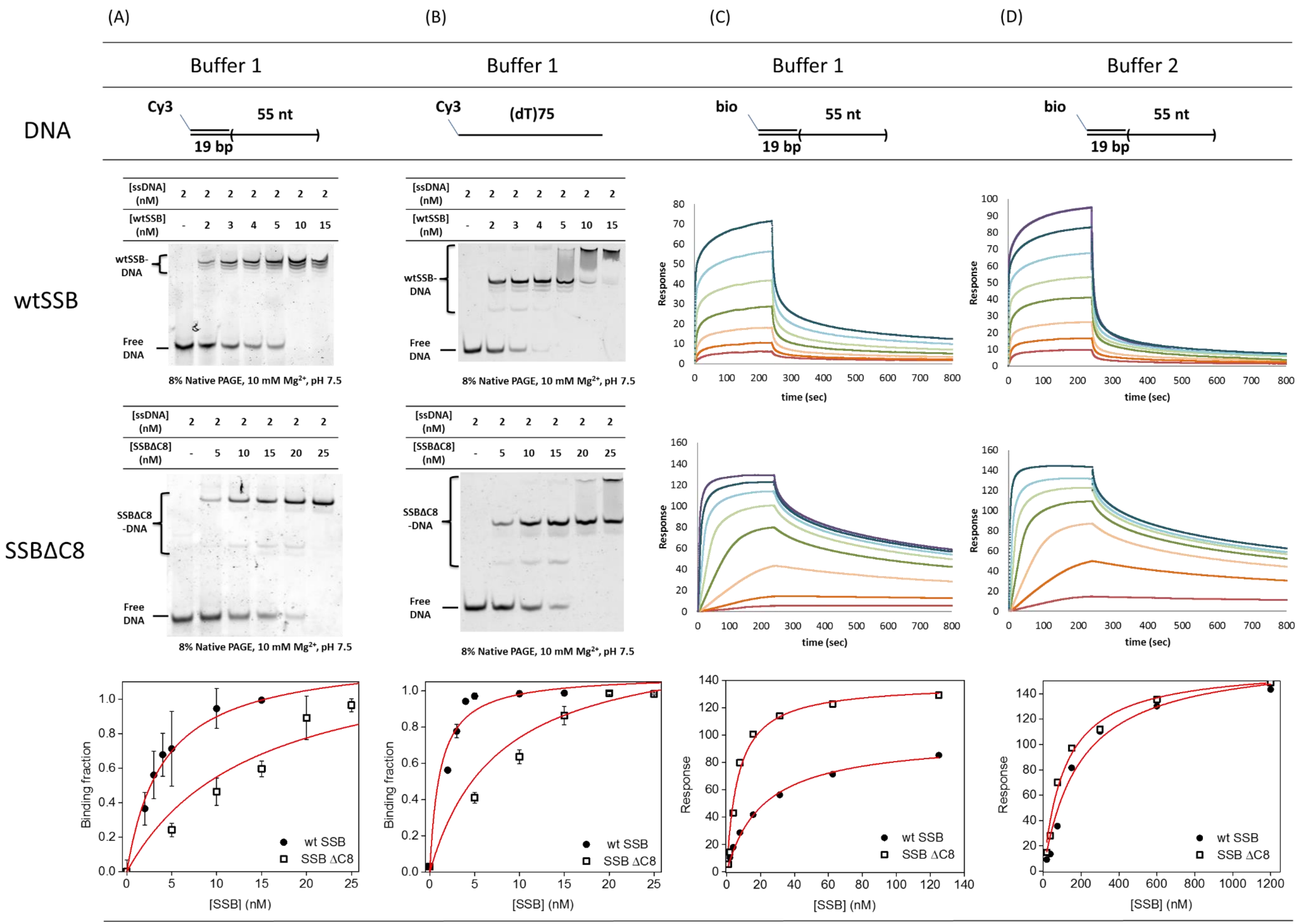

(A)
(B)
(C)
(D)
Buffer 1
Buffer 1
Buffer 1
Buffer 2
DNA
Cy3
55 nt
19 bp
Cy3
(dT)75
bio
55 nt
19 bp
bio
55 nt
19 bp
wtSSB
SSBΔC8
[ssDNA] (nM)
[wtSSB] (nM)
[SSBΔC8] (nM)
wtSSB-DNA
SSBΔC8-DNA
Free DNA
8% Native PAGE, 10 mM Mg2+, pH 7.5
Response
time (sec)
Binding fraction
[SSB] (nM)
wt SSB
SSB ΔC8

Figure S7. Wild-type SSB and SSBΔC8 binding affinities determination using electrophoretic mobility shift assay (EMSA) (A and B) and surface plasmon resonance (SPR) (C and D) experiments under various conditions: (A) using hybrid DNA substrate containing 55 nt ssDNA in buffer 1; (B) using 75 nt ssDNA in buffer 1; using hybrid DNA substrate containing 55 nt ssDNA in (C) buffer 1 and (D) buffer 2. Buffer 1 condition for EMSA is the same used in the single-molecule experiment. SPR experiments were also done in buffer 1 with additional 0.005% of tween-20. In EMSA experiments, the binding fraction determined from integrating all protein-DNA bands as a function of SSB concentrations. For SPR experiments, the binding fraction determined from the response as a function of SSB concentrations. Solid circles are for wtSSB and open squares are for SSBΔC8.

	$(dT)_{70}$	$(dT)_{135}$	$(dT)_{200}$	AC264
$(dT)_{70}$	**1**	0.008	**0.813**	0.028
$(dT)_{135}$		**1**	0.018	**0.677**
$(dT)_{200}$			**1**	0.053
AC264				**1**

Table S1. Pairwise t-test p-values of nucleation times of RecA E38K assembly on different gapped DNA substrates. Data are from Figure 3A.
